# *Listeria monocytogenes* in foods—From culture identification to whole‐genome characteristics

**DOI:** 10.1002/fsn3.2910

**Published:** 2022-05-03

**Authors:** Jacek Osek, Beata Lachtara, Kinga Wieczorek

**Affiliations:** ^1^ Department of Hygiene of Food of Animal Origin National Veterinary Research Institute Puławy Poland

**Keywords:** classical methods, genotyping, identification, *Listeria monocytogenes*, molecular methods

## Abstract

*Listeria monocytogenes* is an important foodborne pathogen, which is able to persist in the food production environments. The presence of these bacteria in different niches makes them a potential threat for public health. In the present review, the current information on the classical and alternative methods used for isolation and identification of *L. monocytogenes* in food have been described. Although these techniques are usually simple, standardized, inexpensive, and are routinely used in many food testing laboratories, several alternative molecular‐based approaches for the bacteria detection in food and food production environments have been developed. They are characterized by the high sample throughput, a short time of analysis, and cost‐effectiveness. However, these methods are important for the routine testing toward the presence and number of *L. monocytogenes*, but are not suitable for characteristics and typing of the bacterial isolates, which are crucial in the study of listeriosis infections. For these purposes, novel approaches, with a high discriminatory power to genetically distinguish the strains during epidemiological studies, have been developed, e.g., whole‐genome sequence‐based techniques such as NGS which provide an opportunity to perform comparison between strains of the same species. In the present review, we have shown a short description of the principles of microbiological, alternative, and modern methods of detection of *L. monocytogenes* in foods and characterization of the isolates for epidemiological purposes. According to our knowledge, similar comprehensive papers on such subject have not been recently published, and we hope that the current review may be interesting for research communities.

## Graphical abstract

In the current review, classical microbiological methods and rapid techniques based on DNA amplification, immunological principles, or biosensor technology used for *Listeria monocytogenes* detection and identification in food are described. Furthermore, molecular‐based approaches such as whole‐genome sequence techniques for characteristics and typing of the isolates for epidemiological purposes have been discussed.

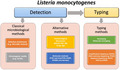

## 1 INTRODUCTION

*Listeria* (*L*.) *monocytogenes* was first described in 1910 when the bacteria, named at that time as *Bacillus hepatis*, were isolated from the liver of a rabbit in Sweden (Carvalho et al., 2014). Similar bacteria causing illness in rabbits and guinea pigs were also identified in the United Kingdom in 1926 and named *Bacterium monocytogenes* (Murray et al., 1926). The bacterium name was then changed to *Listerella hepatolytica* in honor of Joseph Lister, the British pioneer of antiseptic surgical operations (Gray & Killinger, 1966). Finally, in 1940, the definitive name (*L. monocytogenes*) was given (Lamont & Sobel, 2011).

The genus *Listeria* consists of 17 bacterial species, including nine *Listeria* species newly described since 2009 (Orsi & Wiedmann, 2016). All of them are characterized with a low guanine and cytosine DNA content (from 34.6% to 41.6%), but only two species, *L. monocytogenes* and *L. ivanovii*, are considered pathogenic for humans (Bakker et al., 2010; Cummins et al., 1994). There have been also rare reports on *L. seeligeri* isolation from sporadic listeriosis cases (Rocourt et al., 1986). Furthermore, *L. innocua* was initially considered nonpathogenic and nonhemolytic, but recently some strains have been shown to invade human Caco‐2 cells at same levels as *L. monocytogenes* and were virulent in a mouse model (Bakker et al., 2010; Johnson et al., 2004). *L. innocua* has also been at least once identified in a person with fatal listeriosis which may also support that some strains may be able to cause disease (Perrin et al., 2003).

*Listeria monocytogenes* was first isolated from humans in 1929 in Denmark by Nyfeldt, who claimed that these bacteria were the cause of infectious mononucleosis (Nyfeldt, 1937). Then, *L. monocytogenes* was recognized as pathogen that was responsible for sporadic infections in workers contacted with the diseased animals (Lamont & Sobel, 2011).

*Listeria monocytogenes* is a Gram‐positive bacterium of 0.5–4 μm in diameter and 0.5–2 μm in length that is unable to produce spores. The microorganism is facultatively anaerobic, generally motile due to the presence of flagella at temperature range of 22–28°C but nonmotile above 30°C (Allerberger, 2003). These bacteria are usually catalase‐positive; however, catalase negative isolates also have been reported (Cepeda et al., 2006). Furthermore, *L. monocytogenes* strains are oxidase, urea, and indole negative but catalase‐positive and are able to hydrolyze aesculin (Cepeda et al., 2006). The growth temperature for *L. monocytogenes* ranges from −0.4 to 45°C, with an optimum temperature of 37°C. The bacteria can survive at a relatively low water activity (aW <0.90) and a broad pH range between 4.6 and 9.5 as well as have the ability to tolerate salt conditions (NaCl) up to 20% (Buchanan et al., 1989; Bucur et al., 2018). Total inactivation of these bacteria occurs at 75°C (Muskalska & Szymczak, 2015). These growth conditions made these bacteria able to survive and multiply under extreme environmental conditions which are often present at food production facilities (Gray et al., 2006; Ranasinghe et al., 2021). Consequently, *L. monocytogenes* is an important foodborne pathogen that frequently causes sporadic infections or disease outbreaks with significant case numbers and a mortality rate of 20%–30% worldwide (Buchanan et al., 2017). The disease caused by *L. monocytogenes*, called listeriosis, is categorized into two forms: severe invasive listeriosis and noninvasive febrile gastroenteritis (Buchanan et al., 2017). The expression of both forms of the infection depends on several factors, mainly on the age of the infected person, its immune status, infectious dose, and the virulence properties of strain ingested (Poimenidou et al., 2018).

The route of transmission of *L. monocytogenes* to humans was unclear until the 1980s when several outbreaks in the USA and Switzerland indicated that the source of bacteria was food, including dairy products, meat products, seafood products, and vegetables (Klumpp & Loessner, 2013; Lekkas, 2016; Ragon et al., 2008; Zuber et al., 2019). Listeriosis is now believed to be a zoonotic foodborne disease, although other possible routes of infections in humans, such as direct contact with infected animals or contaminated environments, are also possible (Hilliard et al., 2018; Vázquez‐Boland et al., 2001). The infective dose of *L. monocytogenes* to cause listeriosis is difficult to assess, but it has been estimated at 10^4^ to 10^7^ bacteria in susceptible persons to more than 10^7^ in healthy individuals (Angelo et al., 2017; Buchanan et al., 2009; Hoelzer et al., 2013; Pouillot et al., 2014, 2016).

The invasive form of listeriosis mostly occurs in immunocompromised individuals and manifests as sepsis, meningitis, endocarditis, encephalitis, meningoencephalitis, septicemia, and brain infection (Doganay, 2003). Noninvasive listeriosis usually develops in immunocompetent adults, and symptoms of meningitis, septicemia, and febrile gastroenteritis with fever and watery diarrhea lasting for 2–3 days are developed (Mateus et al., 2013). These symptoms are usually self‐limiting and infected persons do not need any medical attention (Allerberger & Wagner, 2010; Doganay, 2003; Swaminathan & Gerner‐Smidt, 2007). The clinical signs of listeriosis often appear after a long incubation time (1–70 days), which has a great influence on epidemiological investigations to trace the source of infection (Buchanan et al., 2017). The incidence of the disease is rather low but hospitalization rate is very high, over 95% (EFSA & ECDC, 2021; Scallan et al., 2011). Listeriosis is especially dangerous for the elderly, pregnant women, unborn babies, and immunocompromised people (Noordhout et al., 2014). In 2019, 2621 confirmed cases of invasive listeriosis in humans were noted in the European Union, with the notification rate of 0.46 cases per 100,000 population (EFSA & ECDC, 2021). Among the cases with information on the hospitalization status, 92.1% were hospitalized and 300 persons died due to *L. monocytogenes* infection (EFSA & ECDC, 2021). In the USA, the Centers for Disease Control and Prevention estimate that about 1600 people get listeriosis each year, and about 260 die. The hospitalization rate of the disease is ca. 94% (www.cdc.gov).

## 2 *L*. *monocytogenes* IN FOOD AND ENVIRONMENTS

*Listeria monocytogenes* is a ubiquitous bacterium and has been mostly identified throughout the environment. It was isolated from soil, water, and feed, although the bacteria are usually found there in a low number (Dhama et al., 2015). It has been described that silages are the most common source of farm environment contamination with *L. monocytogenes* (Gismervik et al., 2015). Many domestic animals, especially ruminants such as goats, cattle, and sheep, carry the bacteria in the intestinal tract and frequently contaminate animal breeding environment with *L. monocytogenes* (Dhama et al., 2015). The ability to survive the microorganisms in the environment, i.e., sewage, river water, and sewage sludge, was demonstrated for at least 8 weeks (Rodríguez‐Campos et al., 2019; Watkins & Sleath, 1981). Thus, there is a potential risk of application of such contaminated material as organic fertilizers which may contribute to subsequent food contamination and human listeriosis development (Schuchat et al., 1991).

Several investigations have shown that *L. monocytogenes* is widely distributed in food processing environments (Carpentier & Cerf, 2011; Ferreira et al., 2014; Tompkin, 2002; Tompkin et al., 1992). It has been demonstrated that the bacteria are able to persist there for a long time due to ineffective cleaning and sanitation, inadequate conditions of food production equipment, or insufficient controls of movement of people (Buchanan et al., 2017). Many *L. monocytogenes* strains are resistant to different food processing conditions, such as low humidity or low oxygen content in food environments. Thus, the presence of the microorganisms in food production plants seems to be a main source of postprocessing contamination (Bucur et al., 2018; Ferreira et al., 2014; Hoelzer et al., 2012; Malley et al., 2015). Some *L. monocytogenes* strains are able to persist for years in food production environments due to either survival and growth in the plant niches which are difficult to clean and disinfect or repeated re‐introduction of such strains by workers or contaminated meat (Ferreira et al., 2014). Persistence of such strains may be contributed by several external factors as poor hygiene practice or ineffective sanitizers but also by the presence of genetic markers in some *L. monocytogenes* strains that are responsible for biofilm production or interactions with native microbiota (Fox et al., 2012; Harter et al., 2017; Lee et al., 2019; Nilsson et al., 2011; Rodríguez‐Campos et al., 2019; Schmitz‐Esser et al., 2015). However, the role of these factors in the persistence mechanisms is still under investigation.

## 3 CULTURE‐BASED DETECTION METHODS

The presence of *L. monocytogenes* in different foods and food processing environments makes the bacteria a potential threat for public health. Therefore, several methods have been applied for their detection and identification based on selective pre‐enrichment, enrichment, and plating on agar plates, followed by the characterization of *Listeria* isolates using conventional microbiological methods such as colony morphology, sugar fermentation, and hemolytic properties (Gasanov et al., 2005). These classical methods are usually very sensitive, standardized, and still used in many laboratories, especially when the bacterial isolate is needed for further characterization or comparison (Table 1).

**TABLE 1:** Advantages and limitations of microbiological, alternative, and molecular methods used for *L. monocytogenes* identification in food

Identification methods	Advantages	Limitations	References
Microbiological methods			
Culture‐based methods: pre‐enrichment (e.g., half‐Fraser broth) enrichment (e.g., Fraser broth) agar plating (e.g., PALCAM agar or ALOA chromogenic agar) bacterial identification (e.g., biochemical tests)	Easy to perform, especially with chromogenic media Cost‐effective Only detection of viable cells Not inhibited by matrix components Approved by regulatory authorities	Time‐consuming and labor‐intensive (5–10 days to confirm a positive sample) Results may depend on environmental conditions Injured, stressed cells may not be detected Possible false‐negative or false‐positive results	Dwivedi and Jaykus (2011), Gasanov et al. (2005), Jadhav et al. (2012), Jeyaletchumi et al. (2010), Kumar et al. (2014), Magalhães et al. (2014), Van Netten et al. (1989), Zunabovic et al. (2011)
Alternative methods			
Immunological methods: ELISA (e.g., TRANSIA™ PLATE *Listeria monocytogenes*) ELFA (e.g., VIDAS^®^ LMO2)	Easy to perform Reproducible Sensitive, especially after enrichment step Can be automated Easily accessible (commercial kits available)	Sensitivity and specificity depend on the quality of antibodies Pre‐enrichment is required to express cell surface antigens Possible false‐negative or false‐positive results May result with cross‐reactivity with closely‐related antigens Presumptive samples need further confirmation Should be validated against microbiological methods	Gasanov et al. (2005), Jasson et al. (2010), Vaz‐Velho et al. (2000)
Biosensors: optical (e.g., Organic Light‐Emitting Diode; OLEL) cell‐based (e.g., BioElectric Diagnostic; B.EL.D) electrochemical (e.g., Carbon Ionic Liquid Electrode; CILE)	Highly sensitive, specific, reproducible, robust Rapid or real‐time detection Many systems are portable and easy to handle Cost‐effective	High cost Results may depend on food matrix Possible field or on‐spot analysis Should be validated against microbiological methods	Arora et al. (2011), Bìberoğlu (2020), Hadjilouka et al. (2020), Li et al. (2021), Silva et al. (2020), Soni et al. (2018), Sun et al. (2012), Turner (2000)
Spectrometry‐based methods: MALDI‐TOF MS (e.g., Microflex LT) VITEK^®^ MS (e.g. VITEK^®^ MS Advanced Expert System^™^)	Rapid Accurate Sensitive	High cost Results may depend on environmental conditions	Angeletti (2016), Araújo et al. (2020), Bastin et al. (2018), De Carolis et al. (2014), Fenselau and Demirev (2001), Ghamisi et al. (2017), Suarez et al. (2013), Wieser et al. (2012)
Molecular methods			
PCR: simple PCR quantitative PCR (qPCR)	Highly sensitive and specific Simple to perform May be automated Reliable results	Sensitivity may depend on PCR inhibitors present in food Require DNA isolation Presumptive samples need further confirmation Identify viable and nonviable cells Should be validated against microbiological methods	Gasanov et al. (2005), Jadhav et al. (2012), Law et al. (2015), Law et al. (2015a), Swetha et al. (2012), Zhao et al. (2014)
Multiplex PCR: quantitative multiplex qPCR	Highly sensitive and specific Detection of different pathogens or species Automated Reliable results	Require DNA isolation Sensitivity may depend on PCR inhibitors present in food Presumptive samples need further confirmation Highly depends on primer design and amplification conditions Identify viable and nonviable cells Requires gel electrophoresis Should be validated against microbiological methods	Law et al. (2015a), Liu et al. (2007), Ryu et al. (2013), Zhao et al. (2014)
Real‐time PCR: simple rt‐PCR (e.g., BAX^®^ System Real‐time PCR Assay *Listeria monocytogenes*) multiplex rt‐PCR quantitative rt‐PCR quantitative multiplex rt‐PCR (e.g., iQ‐Check *Listeria monocytogenes* II Kit)	Higher sensitivity and specificity than simple PCR and multiplex PCR More rapid than simple PCR and multiplex PCR Allows high‐throughput analysis Reproducible Real‐time detection Easily accessible (commercial kits available)	Cost‐related Sensitivity may affect by PCR inhibitors present in food Needs trained personnel Identify viable and nonviable cells Should be validated against microbiological methods	Cady et al. (2005), Garrido‐Maestu et al. (2014), Hage et al. (2014), Heo et al. (2014), Janzten et al. (2006), Law et al. (2015a), Levin (2005), Mackay and Landt (2007), Patel et al. (2006), Zhao et al. (2014)
LAMP: simple LAMP (e.g., MicroSEQ™ *Listeria* spp. Detection Kit) multiplex LAMP (e. g., 3M™ Molecular Detection System) reverse‐transcription LAMP real‐time LAMP (e.g., Loopamp^®^ *Listeria monocytogenes* Detection Kit) in situ LAMP	Simple to perform More rapid than PCR Higher sensitivity and specificity than PCR Less sensitive to potential inhibitors in food Cost‐effective No thermal cycling required May be automated	Complicated primer design Should be validated against microbiological methods	Law et al. (2015a), Law et al. (2015b), Ledlod et al. (2020), Nagamine et al. (2002), Nathaniel et al. (2019), Notomi et al. (2000), Radoshevich and Cossart (2018), Tang et al. (2011), Zhao et al. (2014)
NASBA: RNA amplification (NucliSENS EASYQ^®^) DNA amplification	Sensitive and specific Cost‐effective No thermal cycling required Able to detect viable cells May be automated	Viable microorganisms required Difficulties in handling RNA Should be validated against microbiological methods	Blais et al. (1997), Dwivedi and Jaykus (2011), Guatelli et al. (1990), Li and Macdonald (2015), Nadal et al. (2007)
DNA microarrays (e.g., Listeria GeneChip)	Rapid Highly sensitive and specific Allows high‐throughput analysis Enables detection of multiple pathogens	Cost‐related Trained personnel required Difficult to distinguish viable and nonviable cells	Bang et al. (2013), Gasanov et al. (2005), Govindarajan et al. (2012), Laksanalamai et al. (2012), Law et al. (2015a), Severgnini et al. (2011)

There are many various selective enrichment and plating bacteriological media that are used for the detection and isolation of *L. monocytogenes* from food and food production environment samples. According to several internationally accepted regulations, the conventional isolation methods must be able to detect one *Listeria* organism in 25 g of food (Anon., 2005). To achieve this goal, the food samples must undergo the enrichment step, which allows the bacteria to grow until a detectable level of ca. 10^4^–10^5^ cells per ml before plating the culture on selective media. Since *L. monocytogenes* is a slow‐growing microorganism, in enrichment media antimicrobial agents are used to suppress competing microflora present in analyzed samples. For this purpose, acriflavine, nalidixic acid, and cycloheximide are usually added (Beumer & Hazeleger, 2003; Gasanov et al., 2005; Janzten et al., 2006; Jeyaletchumi et al., 2010; Law et al., 2015). Acriflavine inhibits RNA synthesis and mitochondriogenesis and thus suppresses the growth of Gram‐positive bacteria other than *L. monocytogenes*. Nalidixic acid inhibits DNA synthesis and subsequently prevents the growth of Gram‐positive bacteria, whereas cycloheximide inhibits protein synthesis in eukaryotic cells by binding to 80S rRNA and it is used to prevent the growth of most yeasts and molds (Beumer & Hazeleger, 2003; Janzten et al., 2006). Furthermore, *L. monocytogenes* is able to hydrolyze carbohydrate esculin, which in the presence of iron forms a black phenolic compound derived from the aglucon (Beumer & Hazeleger, 2003). Therefore, esculin is often added to *Listeria* enrichment and plating media, e.g., Fraser broth (Fraser & Sperber, 1988; ISO, 2017). There are also other antimicrobial agents that may be added into the *Listeria* identification media, e.g., broad‐spectrum ceftazidime, moxalactam, lithium chloride (Janzten et al., 2006).

PALCAM and Oxford plating selective media, often used for isolation of *L. monocytogenes*, are recommended by ISO and FDA (ISO, 2017; Law et al., 2015a). The selectivity of PALCAM agar depends on the presence of lithium chloride, polymyxin B, acriflavine, and ceftazidime, whereas its differentiation of the target organism is based on esculin hydrolysis and mannitol fermentation (Magalhães et al., 2014; Van Netten et al., 1989). All *Listeria* spp. are able to hydrolyze esculin; therefore, their colonies are gray‐green in color with a black center and a black halo. If *Enterococcus* sp. or *Staphylococcus* sp. are occasionally present in the microflora of the food sample tested, they may be distinguished from listeria by mannitol fermentation which changes in the colony and/or surrounding medium from gray or red to yellow due to the production of acids. Colonies of the contaminated microflora, which are mannitol fermenting microorganisms, become yellow with a yellow halo or gray with a brown‐green halo (Kumar et al., 2014; Van Netten et al., 1989).

Oxford agar, initially developed for the isolation of *L. monocytogenes* from clinical samples, is also used for the detection of this microorganism from various food samples (Curtis et al., 1989; Pinto et al., 2001). There are several selective components present in the Oxford medium (lithium chloride, acriflavine, colistin sulfate, cycloheximide, cefotetan, fosfomycin) which inhibit growth of potentially present nontarget microflora, whereas the differentiation of *Listeria* sp. is based on esculin hydrolysis, similarly like on PALCAM agar (Curtis et al., 1989; Janzten et al., 2006). On Oxford medium, *L. monocytogenes* colonies are olive‐green with a black halo, and after 48 h of incubation they become darker with a black center and surrounded by black zones. Colonies of other *Listeria* sp. are black with a black halo after 24 h of incubation and they remain the same after 48 h (Curtis et al., 1989; Magalhães et al., 2014).

Both PALCAM and Oxford media are useful for the isolation of *Listeria* sp. from food samples rich in competitive microflora and from processed products, where *Listeria* cells are often damaged or stressed (Law et al., 2015). However, one of the main limitations of PALCAM and Oxford media is their inability to distinguish between pathogenic *L. monocytogenes* from nonpathogenic *Listeria* of other species (Zunabovic et al., 2011).

Although the abovementioned PALCAM and Oxford media contain selective agents to inhibit the growth of most other than *L. monocytogenes* microorganisms, some other bacteria are able to grow and utilize esculin (i.e., *Enterococcus* and *Bacillus*) and may have a similar appearance. Therefore, further tests are required to definitely identify *Listeria* colonies. These microorganisms are Gram‐positive rods, aerobic and facultatively anaerobic, nonspore forming, the vast majority of them are catalase‐positive, and oxidase‐negative (Cepeda et al., 2006). Most strains are motile at 28°C and nonmotile at 37°C (Gasanov et al., 2005). There are commercially available biochemical kits which have been extensively validated and are now used within standard microbiological methodology as ISO (ISO, 2017).

The development of blood‐containing media allowed the separation of the hemolytic *Listeria* species (*L. monocytogenes*, *L. seeligeri*, and *L. ivanovii*) from the nonhemolytic and nonpathogenic *L. innocua*, *L. grayi*, and *L. welshimeri*. This feature of *L. monocytogenes* was utilized for its identification on, e.g., enhanced hemolysis agar (EHA) described by Cox et al. (1991) and further improved by Beumer et al. (1997) or *L. monocytogenes* blood agar (LMBA), especially used after enrichment of the sample (Johansson, 1998). The CAMP (Christie–Atkins–Munch–Peterson) test is also utilized to differentiate between hemolytic and nonhemolytic *Listeria* species (McKellar, 1994). However, this assay sometimes does not correctly differentiate between *L. monocytogenes* and *L. ivanovii* (Vázquez‐Boland et al., 1990). The traditional CAMP test is often replaced by simple and specific commercially available β‐lysin discs (Janzten et al., 2006). Fermentation of D‐xylose and L‐rhamnose can also be used to differentiate *L. monocytogenes* (xylose‐negative and rhamnose‐positive) from the other two hemolytic species *L. ivanovii* and *L. seeligeri* which are xylose‐positive and rhamnose‐negative (Janzten et al., 2006).

A milestone in improvement of *L. monocytogenes* isolation was made when chromogenic media were introduced. One of the first such agars was Agar *Listeria* according to Ottaviani and Agosti (ALOA), described by Ottaviani et al. (1997). This medium contains 5‐bromo‐4‐chloro‐3‐ indolyl‐β‐D‐glucopyranoside (X‐glucoside), a chromogenic compound which is a substrate for the detection of β‐glucosidase, an enzyme that catalyzes the hydrolysis of the glycosidic bonds to terminal nonreducing residues in β‐D‐glucosides and oligosaccharides, with release of glucose (Ottaviani et al., 1997). β‐Glucosidase is common to all *Listeria* species, which appear as green‐blue colored colonies on the ALOA agar plates. The selectivity of the medium is achieved by the addition of lithium chloride and antimicrobials such as ceftazidime, polymyxin B, nalidixic acid, and cycloheximide (Beumer & Hazeleger, 2003; Magalhães et al., 2014).

Further differentiation of *L. monocytogenes* from other *Listeria* on ALOA agar is obtained through the production of a phosphatidylinositol‐specific phospholipase C (PI‐PLC) secreted by *L. monocytogenes*, which hydrolyzes of L‐α‐phosphatidylinositol resulted in green‐blue colonies surrounded by an opaque halo, whereas other *Listeria* spp., lacking phospholipase C enzyme (except some strains of *L. ivanovii*), grow as colonies with a green‐blue color but without the halo (Magalhães et al., 2014; Restaino et al., 1999). An improved ALOA medium—Rapid’L.mono agar—is based also on the same enzyme system, but hydrolysis of a different substrate by PI‐PLC, which is 5‐bromo‐4‐chloro‐3‐indolyl‐myo‐inositol‐1‐phospjhate (X‐IP). On Rapid’L.mono agar, *L. monocytogenes* (and some strains of *L. ivanovii*) are identified as blue colonies (Foret & Dorey, 1997). However, differentiation of *L. monocytogenes* from *L*. *ivanovii* is possible due to the presence of xylose in the medium which is metabolized by the latter species results in the production of blue colonies with a yellow halo, whereas *L. monocytogenes* is not able to metabolize this sugar and its colonies are blue but without halo (Janzten et al., 2006; Zunabovic et al., 2011).

Another chromogenic medium commonly used for *L. monocytogenes* identification is CHROMagar^TM^ *Listeria* (Becton Dickson Diagnostics), one of the variants of ALOA. On this medium, colonies of *L. monocytogenes* appear blue with a white halo, whereas colonies of other *Listeria* spp. are also blue but without halo. It has to be noted that some strains of *L. ivanovii* may also grow on CHROMagar™ *Listeria* as blue colonies with a white halo (Magalhães et al., 2014).

All chromogenic media are useful for rapid isolation and distinguish of *L. monocytogenes* from other nonpathogenic *Listeria* species. However, the specificity of these media may be different in relation to food samples tested (Andritsos et al., 2013). Therefore, for rapid but also specific identification of *L. monocytogenes*, it is recommended to use a combination of selective and chromogenic media based on the laboratory's experience and the sample type to be examined since there is no “gold standard” medium for the isolation of *L. monocytogenes* from various food samples (Andritsos et al., 2013; Churchill et al., 2006).

## 4 ALTERNATIVE DETECTION METHODS

Morphological and biochemical approaches used for identification of *L. monocytogenes* are simple, sensitive, and inexpensive but laborious and time‐consuming as they require more than 7 days for the detection and confirmation of the pathogen (Dwivedi & Jaykus, 2011; Law et al., 2015a). However, most alternative nonmolecular methods still lack sufficient sensitivity and specificity for direct identification of the target microorganisms in samples tested. Therefore, different kinds of foods investigated toward the presence of *L. monocytogenes* need to be enriched using a classical microbiological step before analysis (Janzten et al., 2006).

### 4.1 Immunological assays

There are different kinds of immunoassays which are based on binding of different antibodies (e.g., monoclonal, polyclonal, and recombinant antibodies) to the specific antigen on the surface of *Listeria*. They have been used for many years and are characterized by their simplicity, sensitivity, and accuracy (Gasanov et al., 2005; Jasson et al., 2010). However, to increase their sensitivity, a pre‐enrichment step is needed in order to eliminate the background microflora present in test sample and to increase the number of target *L. monocytogenes* cells (Gasanov et al., 2005; Jasson et al., 2010). The advantage of immunoassays is their easy accessibility since they are available as commercial kits and are approved by food regulatory authorities (Gasanov et al., 2005).

Immunoassay techniques used for detection of *Listeria* usually utilize antibodies directed toward bacterial cell structural components, such as flagella, LLO (listeriolysin) toxin, and protein p60 (invasion‐associated protein encoded by the *iap* gene) (Janzten et al., 2006; Shamloo et al., 2019). The enzyme‐linked immunosorbent assay (ELISA), including its variant enzyme‐linked immunofluorescent assay (ELFA), is one of the most common antibody tests used for *L. monocytogenes* detection, especially in foods. It is an easy and rapid method, but its specificity and sensitivity depend on the quality of the antibody used. The most accurate results are obtained when monoclonal antibodies, which react only with *L. monocytogenes* target‐specific proteins, are applied. An example of ELISA for specific *L. monocytogenes* identification is TRANSIA™ PLATE *Listeria monocytogenes* developed by BioControl Systems. The test based on a two‐step sandwich‐type reaction, where highly specific antibodies for antigens only produced by *L. monocytogenes* are used which eliminate cross reactions with other *Listeria* species. The assay was validated according to the ISO 16140 standard and was certified by AFNOR and NordVal validation bodies. Another commercially available ELISA‐based *L. monocytogenes* identification and confirmation system is VIDAS^®^ LMO2 (bio‐Mérieux), which demonstrates high specificity and sensitivity when used for food testing (Vaz‐Velho et al., 2000). The method was also validated by AFNOR according to the ISO 16140 protocol. The VIDAS^®^ LMO2 assay enables the detection of *L. monocytogenes* antigens using the ELFA method. This approach can be applied to different foods and samples from food production environments as an alternative rapid and specific method to the ISO 11290‐1:2017 standard (ISO, 2017).

### 4.2 Biosensors

Biosensors are analytical devices used for the detection of chemical substances or target microorganisms that combine a biological component with a physicochemical detector (Turner, 2000). Usually, biosensors convert a biological response into an electric signal by a transducer, which may be optical (e.g., UV, bioluminescence, fluorescence), electrochemical, thermometric, piezoelectric, magnetic, or combinations (Sun et al., 2012; Velusamy et al., 2009). The electronic or optical signal generated by biosensors are measured and recorded in proportion to the specific biological interaction between the analyte and the recognition molecule (Turner, 2000). Using biosensors, several different targets can be detected, from small molecular weight proteins to bacterial cells (Bìberoğlu, 2020).

Application of biosensors for identification of bacteria in food has several advantages: many of the systems are portable, easy to handle; thus, they may be used in the field application or on the spot analysis (Table 2). Furthermore, they have many features such as accurate, close to real time, sensitive, specific, reproducible, robust, and do not require highly trained personnel (Turner, 2000). Currently, fiber optical biosensors, which are one of the most popular devices for detection purposes, have been used for identification of *Listeria* spp. and other bacterial pathogens (Arora et al., 2011). They were applied for detection of *L. monocytogenes* in pure culture and in mixture with other bacteria at the level of 10^3^ cells per ml, as well as artificially contaminated with 10^2^ colony forming unit (CFU) per ml of ready‐to‐eat meat products of beef, chicken, and turkey origins after 18 h enrichment (Ohk et al., 2010). Välimaa et al. (2015) demonstrated that the bacterial detection levels were higher than culture‐based methods, but the sensitivity of the biosensors were not as high as DNA amplification methods. Thus, biosensor‐based detection methods still need to be more thoroughly validated (Soni et al., 2018).

**TABLE 2:** Advantages and limitations of methods used for *L. monocytogenes* typing

Typing method	Advantages	Limitations	References
Serological typing: and H antigens polyclonal antisera monoclonal antibodies	Easy to perform Commercial access to antisera	Expensive antisera needed Laborious and time‐consuming A poor discriminatory power Cross‐reactivity with closely‐related strains	Jadhav et al. (2012), Liu (2006)
Molecular serotyping: PCR multiplex PCR	Rapid and sensitive Easy to perform Cost‐effective	Only molecular serogroups are identified Not able to distinguish between all serotypes	Doumith et al. (2004), Kérouanton et al. (2010), Orsi et al. (2011)
Amplification‐based typing: RAPD	Easy to perform Cost‐effective	Low discrimination and reproducibility Conditions‐sensitive Lack of standardized protocol	Caetano‐Annoles et al. (1992), Hadrys et al. (1992), Kumari and Thakur (2014), Lee et al. (2011)
PCR‐SSCP	Rapid	Low discrimination and reproducibility	Shamloo et al. (2019), Saubusse et al. (2007), Wiedmann (2002)
PCR‐RFLP	Easy to perform	Difficult to interpretation	Hashim and Al‐Shuhaib (2019), Liu (2006)
DNA restriction‐based typing: RFLP PFGE	Highly discriminative Web‐available protocols	Laborious and time‐consuming Requires special equipment Requires trained personnel Conditions‐sensitive Online comparable results obtained in different laboratories	Li et al. (2009), Lopez‐Canovas et al. (2019), Swaminathan et al. (2001), Wiedmann (2002)
Sequence‐based typing: MLVA	Simple to perform Rapid Highly discriminative Cost‐effective Obtained results may be stored in database Web‐based analysis platforms available	Lack of standardized protocol	Løvdal et al. (2021), Lunestad et al. (2013), Martín et al. (2017), Nadon et al. (2017)
MLST	Highly discriminative Suitable for epidemiological investigations Results from different laboratories may be stored in databases Web‐based analysis platforms available Does not require specialized reagents or training	Cost‐related Time‐consuming Less discriminatory for isolates of serotype 4b	Anwar et al. (2022), Henri et al. (2016), Jadhav et al. (2012), Ragon et al. (2008), Salcedo et al. (2003), Wang et al. (2012)
NGS	High sensitivity and specificity Enables detection of multiple pathogens Allows high‐throughput analysis Enables analysis of whole genome A broad molecular typing application	Cost‐related Time‐consuming Trained personnel is needed Bioinformatics are required for data analysis	Hurley et al. (2019), Jagadeesan, Baert, et al. (2019), Jagadeesan, Gerner‐Smidt, et al. (2019), Levy and Myers (2016), Lüth et al. (2018), Lüth et al. (2021), Petersen et al. (2020), Yohe and Thyagarajan (2017), Zhong et al. (2021)

On the other hand, Hadjilouka et al. (2020) described a newly developed method that uses a cell‐based biosensor technology and a portable device called Bio Electric Diagnostics (B.EL.D; EMBIO Diagnostics Ltd.) that is able to provide results of *L. monocytogenes* detection within 3 min after 24 h enrichment. The authors conducted the studies with different kinds of food (ready‐to‐eat lettuce salads, milk, halloumi cheese) and the results indicate that the system was able to identify the bacteria in artificially inoculated samples with 90%–98% accuracy. Furthermore, the limit of detection was determined as low as 0.6 log CFU per ml or g in all food types (Hadjilouka et al., 2020). Recently, Jampasa et al. (2021) described an ultrasensitive electrochemiluminescence sensor based on nitrogen‐decorated carbon dots for *L. monocytogenes* identification using a screen‐printed carbon electrode. This method, under optimal parameters, showed a high specificity and sensitivity (1.0 × 10^–1^ CFU/ml). Another biosensor‐based approach for *L. monocytogenes* identification was developed by Li et al. (2021). This method utilizes an electrochemical biosensor for identification of the so called clustered regularly interspaced short palindromic repeats present in bacteria and archaea (E‐CRISPR), combined with recombinase‐assisted amplification (RAA) for ultrasensitive and highly specific detection of several bacterial species. The assay can detect as low as 0.68 aM of genomic DNA and 26 CFU/ml of *L. monocytogenes* in pure culture with no cross‐reactivity with other nontarget bacteria (Li et al., 2021). Thus, all these newly developed biosensor‐based approaches can be used as relatively simple, highly sensitive, and accurate tools for rapid food analysis toward the presence of *L. monocytogenes* (Silva et al., 2020; Sun et al., 2012).

### 4.3 Spectrometry methods

#### 4.3.1 Matrix‐assisted laser desorption ionization‐time of flight mass spectrometry (MALDI‐TOF MS)

MALDI‐TOF MS is a rapid, accurate, sensitive, and valuable identification technique based on the whole‐cell proteome fingerprint of the bacteria (Fenselau & Demirev, 2001; Holland et al., 1996). The technique allows ionization and vaporization of large nonvolatile biomolecules such as intact proteins which generate mostly single‐charged ions. They are then accelerated through an electrostatic field into the high vacuum flight tube until they reach the detector. The time of flight (TOF) required to reach the detector is dependent on the mass and degree of ionization of the proteins, resulting in a spectral profile unique for a given species, composed of peaks ranging usually from 2 to 20 kDa (De Carolis et al., 2014). The profile obtained comprised mainly ribosomal proteins that are expected to be minimally affected by changes in bacterial culture conditions (Wieser et al., 2012). The collected spectra are compared with a reference databank containing a wide variety of bacterial isolates, and the computer software generates a numerical value (score value) based on the similarities between the observed and stored data sets (Wieser et al., 2012). A score value above 2.0 is generally considered to be a valid species‐level identification (Jarman et al., 2000). The MALDI‐TOF MS approach is simple, robust, and takes around 30 min to give a definitive species identification (Wieser et al., 2012).

In recent years, MALDI‐TOF MS has been implemented in routine food laboratories and used for identification and differentiation of *L. monocytogenes* (Araújo et al., 2020; Barbuddhe et al., 2008; Bastin et al., 2018; Jadhav et al., 2014; Jadhav et al., 2015; Karasu‐Yalcin et al., 2021; Li et al., 2022; Ojima‐Kato et al., 2016; Thouvenot et al., 2018). It has been shown that the method was suitable for identification and typing of *Listeria* species as well as for differentiating the isolates at the clonal lineages level (Barbuddhe et al., 2008). MALDI‐TOF MS yielded 100% accuracy for the identification of *L. monocytogenes*, *L. innocua*, *L. ivanovii*, *L. fleischmannii*, *L. grayi*, *L. seeligeri*, *L. weihenstephanensis*, and *L. welshimeri*, as confirmed by whole‐genome sequence analyses (Thouvenot et al., 2018). However, it has been observed that the strain‐level discrimination was influenced by culture conditions (Jadhav et al., 2015). The analysis using this MALDI‐TOF‐MS can be performed directly from bacterial colonies once they are isolated, therefore, reducing the turnaround time for microbial identification (Chen et al., 2017).

The MALDI‐TOF MS technique was also compared with the conventional phenotypic method for routine identification of bacteria to the species level and gave high‐confidence identifications for 639 isolates, of which 635 (99.4%) were correct (Cherkaoui et al., 2010). Furthermore, the MALDI‐TOF MS was compared to the gold standard PFGE method for source‐tracking of the bacteria and both approached demonstrated good congruence with a Wallace coefficient of 0.71 and comparable discriminatory indices of 0.89 and 0.86, respectively (Jadhav et al., 2015). The usefulness of the MALDI‐TOF MS method for rapid, sensitive, and accurate identification of *L. monocytogenes* was demonstrated for different kinds of food and food production environments (Jadhav et al., 2015; Karasu‐Yalcin et al., 2021; Pyz‐Łukasik et al., 2021).

#### 4.3.2 VITEK^®^ Mass Spectrometry (VITEK^®^ MS)

This innovative *L. monocytogenes* identification method is based on the VITEK^®^ (Value, Integrity, Teamwork, Excellence, Knowledge; bioMerieux, Marcy‐l'Étoile, France) principles that use the MALDI TOF MS approach, which provides single‐choice identifications of bacteria at the species, genus, or group level (Rychert et al., 2013; Suarez et al., 2013). Briefly, the VITEK^®^ is an automated microbiology system utilizing growth‐based technology for fast, accurate microbial identification, and antibiotic susceptibility testing (Crowley et al., 2012). The approach uses a fluorogenic methodology for bacteria identification and a turbidimetric analysis for antimicrobial susceptibility testing (AOAC, 2008). The fluorogenic methodology based on the application of chromogenic and fluorogenic substrates enable specific and rapid detection of a variety of bacterial enzymatic activities (AOAC, 2008). The system is available in three formats (VITEK 2 Compact, VITEK 2, and VITEK 2 XL) that differ in increasing levels of capacity and automation. VITEC 2 Compact focuses on the industrial microbiology‐testing environment but also is used in low to middle volume clinical laboratories, whereas VITEK 2 and VITEK 2 XL formats are more intended for clinical microbiology laboratories and provide increased levels of automation for bigger laboratories (Crowley et al., 2012). In the VITEK^®^ system, reagent cards with 64 wells that can each contain an individual test substrate are used. The substrates measure various metabolic activities of bacteria such as acidification, alkalinization, enzyme hydrolysis, and growth in the presence of inhibitory substances. There are currently four reagent cards available for the identification of different organisms: GN for Gram‐negative fermenting and nonfermenting bacilli, GP for Gram‐positive cocci and nonspore‐forming bacilli, YST for yeasts and yeast‐like organisms, and BCL for Gram‐positive spore‐forming bacilli. Identification cards are inoculated with microorganism suspensions using an integrated vacuum apparatus and incubated at 35.5 ± 1.0°C. During incubation, each test reaction is read every 15 min to measure either turbidity or colored products of substrate metabolism. A transmittance optical system allows interpretation of test reactions using different wavelengths in the visible spectrum. Calculations are performed on raw data and compared to thresholds to determine reactions for each test. Test data from an unknown organism are compared to the respective database to determine a quantitative value for proximity to each of the database taxa.

The VITEK^®^ system has been shown in a broad collaborative study as an acceptable automated method for the rapid identification of selected Gram‐positive bacteria, including *L. monocytogenes* (Crowley et al., 2012; Reis et al., 2022; Thomas & Duse, 2019). However, there are also information that although Vitek 2 is a generally reliable method, some *L. monocytogenes* were misidentified as *L. innocua* (De Lappe et al., 2014). Furthermore, it has also been recently shown that the VITEK^®^ 2 automated system could not discriminate atypical strains of the *Listeria* genus and complementary tests, such as PCR and chromogenic media, for the correct identification of these strains were needed (Reis et al., 2022; Thomas & Duse, 2019).

The VITEK^®^ combined with mass spectrometry (VITEK^®^ MS) system reads each spectrum as a series of peaks that are detected and sorted by mass and intensity. With the use of the Advanced Spectra Classifier, better discrimination is provided as every peak is considered in the calculation of the identification result (Ghamisi et al., 2017). It has been shown that VITEK^®^ MS system has the great potential for identification of the majority of pathogens isolated in the clinical microbiology laboratory as well as those potentially present in food, e.g., *L. monocytogenes* (Jamal et al., 2014).

### 4.4 Molecular detection methods

#### 4.4.1 Polymerase chain reaction (PCR)

Molecular methods for detection of *L. monocytogenes* in food and food production environments have been used for many years as an alternative to classical culture procedures (Gasanov et al., 2005). This approach is based on identification of target‐specific DNA sequences with PCR, multiplex PCR (mPCR), quantitative PCR (qPCR), multiplex qPCR, real‐time PCR, loop‐mediated isothermal amplification (LAMP), DNA microarray, and next‐generation sequencing (NGS) (Janzten et al., 2006; Law et al., 2015a; Law et al., 2015; Matle et al., 2020; Notomi et al., 2000; Wang et al., 2020; Witte et al., 2016; Zhu et al., 2015). However, although molecular methods are very sensitive, they are often inhibited by different components present in food. Furthermore, DNA amplification approaches are not able to distinguish living from dead cells of the target bacteria. Thus, enrichment is needed to dilute potential inhibitors and to multiply live microorganisms (Janzten et al., 2006). Molecular methods also require specialized instruments and highly trained personnel (Gasanov et al., 2005; Law et al., 2015b).

Several PCR assays have been used for the detection of bacterial pathogens in foods, including *L. monocytogenes* (Law et al., 2015b; Levin, 2003). This method requires two single‐stranded synthetic oligonucleotides (specific primers) for the amplification of a specific target DNA sequence with a thermostable polymerase during a three‐step process using a thermal cycler. The PCR amplification products are separated by agarose gel electrophoresis and visualized with a DNA stain (e.g., ethidium bromide or SYBR™ Green). Detection of *L. monocytogenes* using PCR is usually carried out after selectively enriching samples for 24–48 h (Gasanov et al., 2005). Several target sequences for the specific detection of *Listeria* spp. and *L. monocytogenes* were selected (Gasanov et al., 2005; Law et al., 2015; Levin, 2003). The PCR primers are often based on the highly conserved 16S rRNA sequence present in all *Listeria* (Somer & Kashi, 2003; Wang et al., 1992). Then, *L. monocytogenes* can be differentiated from other *Listeria* species either by detection of sequence differences within the amplified 16S rRNA gene or by identification of the virulence genes present only in *L. monocytogenes* (Law et al., 2015a, 2015b; Levin, 2003). Several such pathogenic molecular markers have been identified in *L. monocytogenes* and targeted for the PCR detection, e.g., *hlyA* gene codes for listeriolysin O (Aznar & Alarcón, 2003; Deneer & Boychuk, 1991), *iap* gene codes for an invasion‐associated protein known as p60 (Aznar & Alarcón, 2003; Swetha et al., 2012), or *actA* gene responsible for production of a surface protein ActA, playing a role in cell invasion (Levin, 2003; Moriishi et al., 1998). One of the most common targeted *L. monocytogenes* specific sequences for the PCR detection is the *hly*A gene, which encodes for a protein responsible for the pore‐forming activity (listeriolysin O) (Aznar & Alarcón, 2003; Jadhav et al., 2012). It has been found that all clinical *L. monocytogenes* isolates possess the hemolytic activity encoded by the *hlyA* gene; thus, it is a suitable molecular marker for identification of the pathogenic strains (Aznar & Alarcon, 2002; Border et al., 1990; Johnson et al., 1992).

#### 4.4.2 Multiplex PCR

In multiplex PCR (mPCR), the simultaneous amplification of more than one target genes is performed (Law et al., 2015a). Although the basic principles of mPCR are very similar to conventional PCR, several important factors must be taken into account to design a specific and accurate multiplex assay. One of them is primersʼ design, their concentration in the reaction mixture, and similar annealing temperature which are critical for reliable amplification products (Zhao et al., 2014). Other important elements are the PCR buffer, deoxynucleotide, magnesium chloride, and template concentrations as well as temperatures during the amplification steps (Law et al., 2015b; Markoulatos et al., 2002). Using a multiplex PCR, it is possible to detect several virulence‐associated genes of *L. monocytogenes* or to simultaneously discriminate *Listeria* spp. by targeting different genes for each species in a single PCR tube (Ryu et al., 2013). For example, Cooray et al. (1994) developed mPCR with a set of primers targeting three virulence‐associated genes of *L. monocytogenes* (*prfA, hlyA,* and *plcB*), which was successfully used for the pathogen identification in milk. Liu et al. (2007) established another mPCR directed toward the *inlA*, *inlC,* and *inlJ* genes for species‐specific and virulence‐specific determination of *L. monocytogenes*.

#### 4.4.3 Real‐time PCR

The main difference between conventional PCR and real‐time PCR or quantitative PCR (qPCR) is that the latter method does not require gel electrophoresis for the identification of DNA amplification products. The technique is able to continuously monitor the PCR products formation during the reaction by measuring the fluorescent emission signal produced by specific dual‐labeled probes or intercalating dyes (Cady et al., 2005; Law et al., 2015a; Mackay & Landt, 2007). The fluorescence intensity is proportional to the amount of PCR amplicons; therefore, it is possible to follow the amplification in real time, without laborious and time‐consuming gel electrophoresis (Omiccioli et al., 2009; Zhao et al., 2014). Several fluorescent dyes may be used in the real‐time PCR technique, and among them SYBR^®^ Green is the most widely applied double‐strand DNA‐specific dye which binds to the minor groove of the DNA double helix (Gudnason et al., 2007). Upon intercalation with dsDNA SYBR^®^ Green, fluorescence increases up to 1000‐fold (Zipper et al., 2004). Although SYBR^®^ Green exhibits a very strong fluorescent signal, it also inhibits the PCR reaction and has a narrow dynamic range and lower reproducibility compared to other fluorescent dyes (Gasparic et al., 2010; Zipper et al., 2004). Nevertheless, real‐time PCR with SYBR^®^ Green is highly sensitive, able to detect trace amounts of target DNA, and can be automated which avoids the manipulation of the PCR products after amplification, thus reducing any risk of false‐positive results due to possible cross‐contamination between amplification products (Norton, 2002).

There are several alternatives to SYBR^®^ Green dye in real‐time approach, e.g., TaqMan^®^ probes developed by Applied Biosystems^TM^ (currently Thermo Fisher Scientific). The probes are oligonucleotides that contain a fluorophore as the reporter dye covalently attached to the 5′‐end and the quenching dye at the 3′‐end (Hein et al., 2001; Levin, 2005). Several different fluorophores (e.g., 6‐carboxyfluorescein; FAM, or tetrachlorofluorescein; TET) and quenchers (e.g., tetramethylrhodamine; TAMRA) are used (Kutyavin et al., 2000; Levin, 2005). TaqMan^®^ probe is complementary to a specific nucleotide sequence in one of the strands of amplicon internal to both primers, and the system depends on the exonuclease activity of *Taq* DNA polymerase that cleaves a dual‐labeled probe during hybridization to the complementary target sequence and generates fluorophore‐based detection signal (Patel et al., 2006).

Another alternative to SYBR^®^ Green dye or TaqMan^®^ probe in real‐time PCR is molecular beacon which is a probe with hairpin/stem‐and‐loop configuration, in which the sequence complementary to a target sequence is present in the loop portion (Levin, 2005; Patel et al., 2006). Molecular beacons hybridize with template DNA during annealing and undergoing a spontaneous conformational change that separates the two dyes which allow to produce the fluorescent signal directly, without an exonuclease activity of polymerase which is essential for the TaqMan^®^ probe (Leone et al., 1998; Levin, 2005).

Real‐time PCR method was used for *L. monocytogenes* detection in a variety of kinds of foods (Berrada et al., 2006; Garrido‐Maestu et al., 2014; Heo et al., 2014; Kačániová et al., 2015; Kim & Cho, 2010; Köppel et al., 2021; Law et al., 2015a; Liu et al., 2012; O’Grady et al., 2008; Rodríguez‐Lázaro et al., 2004). Different target genes were selected to specifically identify *L. monocytogenes* and to differentiate the pathogen from nonpathogenic other *Listeria* species. One of them was the *actA* gene, responsible for the expression of the major virulence determinant ActA which is necessary for actin polymerization and intracellular motility and cell‐to‐cell spread of the microorganism. This target gene was used for the identification and quantification of *L. monocytogenes* in food with 5′‐nuclease real‐time PCR (Oravcová et al., 2005; Travier et al., 2013). Detection of *L. monocytogenes* in fresh produce using molecular beacon‐qPCR approach targeting the *hlyA* gene responsible for production of listeriolysin O was first described by Liming et al. (2004). The same target marker was applied in four qualitative SYBR green qPCR assays for the detection and discrimination of *Listeria* spp. and *L. monocytogenes* (Barbau‐Piednoir et al., 2013). Another real‐time PCR approach, based on 5′ exonuclease multiplex qPCR and TaqMan^®^ probe, was successfully used for the identification of six *Listeria* species, including *L. monocytogenes* (Hage et al., 2014). Identification of *L. monocytogenes* in food with molecular beacon‐qPCR targeting the *hlyA* gene was also described by Liming et al. (2004).

Currently, several commercial real‐time based kits for *L. monocytogenes* detection are available (Janzten et al., 2006). These include BAX^®^ System Real‐time PCR Assay *Listeria monocytogenes* (DuPont‐Qualicon), approved already in 2002 by USDA as a screening method for *L. monocytogenes* detection in enriched meat and poultry samples (USDA, 2002). The BAX^®^ system for *L. monocytogenes* was also certified by AFNOR (Association Française de Normalisation) and compared with the standard culture methods in a collaborative study which concluded that this system performed well or better than the standard reference methods for *L. monocytogenes* identification (Silbernagel et al., 2004).

There are also many other real‐time PCR assays available on the market for the detection, quantification, and differentiation of *L. monocytogenes* from other species of *Listeria*. The examples of these commercial kits include AmpliTest *Listeria monocytogenes* (real‐time PCR) (Amplicon Sp. z o. o), iQ‐Check *Listeria monocytogenes* II Kit (Bio‐Rad Laboratories), foodproof^®^ *Listeria* plus *L. monocytogenes* Detection LyoKit (BIOTECON Diagnostics GmbH), PCR‐*Listeria monocytogenes* Detection Kit (BioVision, Inc.), *Listeria monocytogenes* Real‐time PCR Kit, RUO (Nzytech), GeneVision^®^ Rapid Pathogen Detection System for *Listeria monocytogenes* (Warnex), Cycleave PCR^®^ *Listeria monocytogenes* (*inlA* gene) Detection Kit (TaKaRaBio, Inc.), and many others.

#### 4.4.4 Loop‐mediated isothermal amplification (LAMP)

Loop‐mediated isothermal amplification is a variant of nucleic acid amplification method which provides a rapid DNA amplification under isothermal conditions (60–65°C) for rapid (even <1 h), sensitive, and specific detection of target microorganisms, including *L. monocytogenes* (Notomi et al., 2000). The main advantages of the LAMP assay if compared to PCR are the use of isothermal amplification and that no complex thermal cyclers are needed (Nagamine et al., 2002). Furthermore, LAMP assay is not sensitive to inhibitors often present in food samples, which affects DNA amplification with thermostable polymerase in the PCR method (Kaneko et al., 2007). During the LAMP reaction, DNA amplification is carried out by *Bst* polymerase, with four primers comprising two inner primers and two outer primers that are used to target six specific regions of target DNA (Law et al., 2015a). The basis LAMP reaction principle is binding of a large amount of pyrophosphate ion by‐product to magnesium ions which results in a white precipitate of magnesium pyrophosphate (Nagamine et al., 2002). The intensity of the precipitate turbidity correlates with the DNA yield obtained during isothermal amplification and it can be assessed visually with a turbidometer or detected by agarose gel electrophoresis with, e.g., SYBR Green I dye (Kaneko et al., 2007; Mori et al., 2001). The amount of amplicons produced by LAMP within 60 min is usually 10^3^‐fold or higher as compared to conventional PCR (Zhao et al., 2014). This is due to amplification performed with four primers targeting six specific DNA regions, which resulted in greater yield of the products and lower detection limits than PCR (Law et al., 2015b).

Several different types of LAMP assays have been developed for the detection of foodborne pathogens, e.g., multiplex LAMP, reverse‐transcription LAMP, real‐time LAMP, and in situ LAMP (Chen et al., 2008; Cho et al., 2014; Han & Ge, 2010; Shao et al., 2011; Tang et al., 2011; Wang et al., 2011, 2020; Ye et al., 2011). It has also been shown that LAMP‐based assays have higher sensitivity (10 to 100 times) than conventional PCR in the identification of bacteria, including *L. monocytogenes*, in various kinds of food (Nathaniel et al., 2019; Wan et al., 2012).

Different *L. monocytogenes* genes were target with primers used in the LAMP assays. One of the most common markers identified is the *hlyA* gene region, responsible for production of listeriolysin O, the important determinant of virulence which is present in the pathogenic strains (Hamon et al., 2012; Radoshevich & Cossart, 2018). This gene was amplified during the identification of *L. monocytogenes* in chicken meat (Tang et al., 2011), raw meat, vegetables, seafood, different kinds of ready‐to‐eat foods of plant and meat origin (deli foods) (Shan et al., 2012), and dairy products (Tirloni et al., 2017). There are also other virulence genes such as *pfrA*, *iap*, *lmo2234*, *lmo0737*, which were successfully used for detection of *L. monocytogenes* with the LAMP approach (Costa et al., 2014; Nathaniel et al., 2019; Wang et al., 2015; Wu et al., 2014). A variant of conventional LAMP assay described by Wu et al. (2014) contained primers specific for the *hlyA* and *iap* genes of *L. monocytogenes*. This double LAMP (dLAMP) was successfully used for the detection of these bacteria in different food samples and it was more rapid, sensitive, specific, and less time‐consuming as compared to normal LAMP assay (Wu et al., 2014). There are also LAMP connected with real‐time monitoring of the amplification products by a turbidimeter platform (LAMP‐turbidity) which eliminates the need for DNA staining and gel electrophoresis and the results are determined automatically via an amplification curve within 1 h (Wachiralurpan et al., 2017). Recently, Ledlod et al. (2020) described a duplex lateral flow dipstick (DLFD) test combined with LAMP for the identification of *Listeria* spp. and *L. monocytogenes* within approximately 45 min. Under the optimized conditions, the detection limits of the approach were 900 femtograms (10^–15^ g; fg) of pure DNA and 20 CFU/ml, respectively. The method demonstrated 100% accuracy when compared to other detection methods, such as ISO11290‐1, ELFA, VIDAS, and PCR (Ledlod et al., 2020).

Loop‐mediated isothermal amplification for the detection of *L. monocytogenes* has also been available commercially, for instance, Loopamp^®^ *Listeria monocytogenes* Detection Kit (Eiken Chemical, Co., Ltd.), Isothermal Master Mix (OptiGene Ltd.), and 3M™ Molecular Detection System (3M Co.).

#### 4.4.5 Nucleic acid sequenced‐based amplification (NASBA)

Nucleic acid sequenced‐based amplification is a transcription‐based amplification system used for the specific replication of nucleic acid sequences under isothermal conditions (Guatelli et al., 1990). During NASBA, amplification of RNA is performed and the obtained single‐stranded RNA is then converted into complementary DNA (cDNA) by the reverse transcriptase (Chan & Fox, 1999; Compton, 1991; Cook, 2003). Amplification of RNA involves two specific primers and three enzymes: reverse transcriptase of avian myeloblastosis virus, T7 RNA polymerase, and RNase H (Guatelli et al., 1990). The forward primer used in NASBA is approximately 45 bases in length and its 5′ end contains a promoter sequence that is recognized by the T7 RNA polymerase (Compton, 1991). After the T7 promoter, there is a 20‐base sequence that is complementary to the target RNA sequence. The reverse primer usually has 20 bases and contains nucleotides identical to the target RNA sequence. The whole reaction requires temperature of 65°C for the annealing of the forward primer at the beginning whereas the remaining amplification step is performed at 41°C (Compton, 1991). The three enzymes used in NASBA are thermolabile and must be added after the annealing step (Deiman et al., 2002).

Nucleic acid sequenced‐based amplification is a very rapid diagnostic method and requires only four to five cycles to achieve up to approximately 10^6^–10^9^ amplified RNA copies within 1.5 h and does not require any specialized equipment to perform the nucleic acid amplification (Compton, 1991; Li & Macdonald, 2015).

Nucleic acid sequenced‐based amplification may also be used for amplification of DNA, but the first denaturation step at 95°C is required to allow the forward primer to bind to single‐stranded DNA. Subsequently, extension is performed with avian virus myeloblastosis reverse transcriptase, followed the second denaturation which enables reverse primer to bind. Since the denaturation step inactivates reverse transcriptase, this enzyme has to be added again together with the remaining two enzymes: T7 RNA polymerase and RNase H (Deiman et al., 2002). Afterward, the final phase of DNA NASBA is identical to that of RNA NASBA, and the obtained amplification product is single‐stranded RNA complementary to the target DNA strand (Voisset et al., 2000). NASBA DNA is usually performed with primers that are directed to easily accessible DNA regions such as bacterial plasmids or low‐melting point sequences (Voisset et al., 2000).

Products obtained after NASBA, which are mainly single‐stranded RNA, were initially detected by agarose gel electrophoresis under denaturating conditions followed by ethidium bromide staining, although it does not stain RNA as efficiently as DNA (Fakruddin et al., 2012; Jean et al., 2002; Nadal et al., 2007). However, to increase the specificity of the method, a confirmatory step is usually required. This involves probe hybridization, enzyme‐linked gel assay, electrochemiluminescence, fluorescence correlation spectroscopy, and NASBA‐coupled molecular beacon for real‐time detection (van Gemen et al., 1994; Leone et al., 1998; Oehlenschlager et al., 1996; Samuelson et al., 1998; Tyagi & Kramer, 1996). The later detection method is able to detect viable microorganisms and to distinguish viable from nonviable bacterial cells through mRNA amplification and the detection of RNA targets which is important in food testing (Blais et al., 1997; Dwivedi & Jaykus, 2011; Nadal et al., 2007; Simpkins et al., 2000).

A specific NASBA system for the detection of *L. monocytogenes*, based on the *hlyA* mRNA sequences, was developed by Blais et al. (1997). The assay was able to detect low numbers of the bacteria (<10 CFU/g) in artificially contaminated dairy and egg products after 48 h enrichment with the 92.6% of specificity. Another NASBA assay coupled with molecular beacon‐based real time was also used for the detection of the *hlyA* gene of *L. monocytogenes* in cooked ham and smoked salmon (Nadal et al., 2007). The method was also able to distinguish viable from nonviable bacterial cells.

*Listeria monocytogenes* was also detected with NASBA using16S rRNA sequences as the target (Uyttendaele et al., 1995). The method was also compared with a modified FDA culture method for detection of *L. monocytogenes* in foods, using 25 g food samples and enrichment for 48 h prior to NASBA detection, and the obtained results were identical for both methods (Uyttendaele et al., 1995).

A NASBA kit directed to *L. monocytogenes* detection has been manufactured by bioMerieux (NucliSENS EASYQ^®^). It is an automated system that combines NASBA amplification and real‐time detection with molecular beacons (Nadal et al., 2007).

#### 4.4.6 DNA microarrays

The DNA microarray technique was initially being applied for the gene expression in bacterial microorganisms, but subsequently it has been used for the detection of foodborne pathogens and investigations performed during epidemiological investigations (Gasanov et al., 2005; Severgnini et al., 2011). In brief, DNA microarrays comprise hundreds of chemically synthesized or PCR‐made oligonucleotide probes (with sequence length ranges from 25 to 80 base pair (bp) which are fluorescently marked and coated on to glass slides or chips (Severgnini et al., 2011). They are arranged in rows and columns for easy identification of the location on the array. Each of the probes is able to target a specific part of the gene sequence which is being identified (Govindarajan et al., 2012). After hybridization, the fluorescent signal is produced from the probe–sample complex, and its intensity is proportional to the concentration of each labeled nucleic acid fragment (Lauri & Mariani, 2009). The fluorescence emission is usually identified automatically by passing a laser beam and fluorescence emission pattern followed by DNA identification performed with a computer (Govindarajan et al., 2012). The microarray technique is very rapid, sensitive, specific, and allows for the identification of several DNA fragments simultaneously (Govindarajan et al., 2012; Law et al., 2015a).

DNA microarray, based on the *iap*, *hly*, *inlB*, *plcA*, *plcB,* and *clpE* genes, was successfully used for simultaneous identification and differentiation of six *Listeria* species, including *L. monocytogenes*, *L. ivanovii*, *L. innocua*, *L. seeligeri*, *L. grayi,* and *L. welshimeri* (Volokhov et al., 2002). Bang et al. (2013) developed and validated a DNA microarray assay which showed 98%–100% and 7%–85% positive hybridization signals for the *L. monocytogenes* 16 strains and for 9 strains of other *Listeria* spp., respectively. The test was then used for the detection of the pathogen in milk and displayed the detection limit of approximately 8 log CFU/ml. The microarrays were also applied for the successful identification and discrimination of *L. monocytogenes* in food or other matrixes by other authors (Borucki et al., 2004; Call et al., 2003; Hmaïed et al., 2014; Laksanalamai et al., 2012; Severgnini et al., 2011; Suo et al., 2010;).

## 5 *L. monocytogenes* TYPING METHODS

### 5.1 Serological typing

*Listeria monocytogenes* is a diverse species, has a ubiquitous nature, and is present in the environment and potentially in different foods. Therefore, differentiation of the isolates and tracking of strains responsible for, e.g., foodborne listeriosis require highly discriminatory typing systems, mainly utilizing molecular approaches (Law et al., 2015b). The classical serotyping method, based on somatic O and flagellar H antigens of *L. monocytogenes*, has a poor discriminatory power, requires specific antisera, and isolates from foods and from environmental sources are frequently nontypable (Liu, 2006). However, this approach allows identification of 13 different serovars, named also serotypes (1/2a, 1/2b, 1/2c, 3a, 3b, 3c, 4a, 4b, 4ab, 4c, 4d, 4e, and 7), based on the combination of O and H antigens (Allerberger, 2003; Seeliger & Langer, 1989). All *L. monocytogenes* serovars are potentially pathogenic for humans, but the vast majority of infections (over 95%) is due to strains belonging to three serotypes 1/2a, 1/2b, and 4b, with serovar 4b responsible for higher hospitalization rates and deaths (Swaminathan & Gerner‐Smidt, 2007).

*Listeria monocytogenes* serotyping based on ELISA was also developed, but it is time‐consuming and requires the access to high quality of specific antisera; thus, this assay is not routinely used for the isolate differentiation (Palumbo et al., 2003).

### 5.2 Molecular serotyping

Classical serotyping of *L. monocytogenes* is laborious and requires high‐quality antisera which are difficult to produce or commercially purchase (Matle et al., 2020). Therefore, during the last years, it has been commonly replaced by rapid molecular serotyping based on PCR (Doumith et al., 2004; Kérouanton et al., 2010). In this method, the *prs*, *ORF2110*, *ORF2819*, *lmo1118*, and *lmo0737* DNA sequences are amplified, which resulted in the products of diverse size (from 370 bp to 906 bp). However, there is a limitation of this assay which is not able to distinguish between serotypes 1/2a and 3a, 1/2c and 3c, 1/2b, 3b and 7, 4a and 4c, 4b, 4d and 4e, but *L. monocytogenes* classified as 3a, 3c, 3b, 7, 4a, 4c, 4d, and 4e are rarely isolated from human clinical listeriosis (Dhama et al., 2015; Orsi et al., 2011). Nevertheless, this molecular serotyping approach has been validated and is now internationally recognized.

After serotyping, *L. monocytogenes* of all serotypes can be further classified into four lineages named as I, II, III, and IV (Kérouanton et al., 2010; Orsi et al., 2011). Lineage I (with serotypes 1/2b and 4b) and lineage II (including serotype 1/2a and other serovars) are mostly responsible for human infections (Kérouanton et al., 2010; Orsi et al., 2011; Ward et al., 2004). The isolates of serotypes 1/2b and 4b within lineage I possess the *Listeria* pathogenicity island 3 with the gene encoding listeriolysin S, hemolytic and cytotoxic virulence factor, which is not present in strains of other lineages (Cotter et al., 2008). Strains of serovars 1/2a, 1/2c, 3a, and 3c, classified to lineage II, often harbor plasmids that are responsible for *L. monocytogenes* resistance to heavy metals (Dhama et al., 2015). Strains of lineages III and IV are rarely isolated, possess distinct genetic and phenotypic characteristics, and are mainly identified in ruminants (Camargo et al., 2016).

## 6 MOLECULAR TYPING

*Listeria monocytogenes* classical and molecular serotyping approaches possess a limited discriminatory, although it is widely used for the rapid screening of *L. monocytogenes* strains during the investigation of source of isolates and their relatedness (Camargo et al., 2016). However, for epidemiological purposes, several typing methods based on the molecular approaches have been introduced for *L. monocytogenes* differentiation (Burall et al., 2016; Camargo et al., 2016; Destro et al., 1996; Gasanov et al., 2005; Haase et al., 2014; Law et al., 2015a; Law et al., 2015; Louie et al., 1996; Matle et al., 2020; Moura et al., 2016; Ruppitsch, Pietzka, et al., 2015; Shamloo et al., 2019).

### 6.1 Amplification‐based typing methods

There are typing methods utilizing DNA amplification that are still used for *L. monocytogenes* differentiation. One of them is random amplification of polymorphic DNA (RAPD), in which a random primer of arbitrary nucleotide sequence is used for amplifying the bacterial DNA fragments, usually within the 0.5–5 kb size range (Caetano‐Annoles et al., 1992; Hadrys et al., 1992). The obtained amplicons are separated by agarose gel electrophoresis, and their polymorphisms are identified after ethidium bromide staining. Unlike traditional PCR analysis, RAPD does not require any specific knowledge of the DNA sequence of the target organism since the primers will or will not amplify a fragment of bacterial DNA, depending on positions that are complementary to their sequence (Williams et al., 1990). The main advantage of RAPD assay is that it is quick, easy to perform, and requires only a low quantities of template DNA. On the other hand, the method possesses a low reproducibility and highly standardized experimental procedures are needed to overcome its sensitivity to the reaction conditions (Kumari & Thakur, 2014). Thus, problems of reproducibility of the RAPD method make it unsuitable for comparison with the results obtained in other laboratories (Kumari & Thakur, 2014). Despite of these limitations, the RAPD approach was widely used for typing of *L. monocytogenes* (Kang et al., 2016; Lee et al., 2011; MacGowan et al., 1993; Zeinali et al., 2015, 2017).

Another approach for *L. monocytogenes* typing based on PCR is DNA fragmentation or conformational variation in PCR products (Shamloo et al., 2019). One of the most common variants of this approach is the PCR‐single strand conformation polymorphism (SSCP) analysis (Duthoit et al., 2003; Orita et al., 1989). This method is based on the principle that specific regions of genomic sequences can be efficiently labeled and amplified simultaneously with labeled substrates during PCR (Wiedmann, 2002). The PCR product is then denatured and resolved by polyacrylamide gel electrophoresis, where mobility of single‐stranded nucleic acid depends not only on its size but also on its sequence. The overall procedure is rapid, simple, and does not require any restriction enzyme digestion, blotting, or hybridization to labeled probes (Orita et al., 1989). In PCR‐SSCP analysis, changes in several hundred bases are detected; thus, it is much more sensitive to the replication errors that occur during the PCR (Orita et al., 1989; Wiedmann, 2002). It has been shown that PCR‐SSCP method, using sequences in the *hlyA* gene, is useful in identification and differentiation of *L*. *monocytogenes* as well as discrimination the pathogenic strains from nonpathogenic *L. innocua*, which is frequently isolated from food (Destro et al., 1996; Duthoit et al., 2003; Manzano et al., 1997; Saubusse et al., 2007).

PCR‐RFLP (PCR‐restriction fragment length polymorphism) typing method is based on PCR amplification one or more *L. monocytogenes* virulence (e.g., *hlyA*, *actA*, *inlA*) or housekeeping (e.g., *16S rRNA*, *23S rRNA*) genes, digestion of the obtained products with restriction endonuclease(s) (e.g., *Hha*I, *Sac*I, *Hinf*I), and separation of the DNA fragments of various sizes by agarose gel electrophoresis (Botstein et al., 1980; Hashim & Al‐Shuhaib, 2019). The main advantage of the method is its simplicity, whereas a limitation is production of several short restriction fragments, making clear separation of the fragments difficult on a gel (Hashim & Al‐Shuhaib, 2019). PCR‐RFLP was used for differentiation of *L. monocytogenes* subtypes and to track the strains during epidemiological investigations (Liu, 2006; Paillard et al., 2003; Rousseaux et al., 2004; Wiedmann et al., 1997).

### 6.2 DNA restriction‐based typing methods

#### 6.2.1 Restriction fragment length polymorphism (RFLP)

Restriction fragment length polymorphism was one of the first and easiest molecular methods used for typing of bacteria which is based on the detection of variations in DNA sequence (Li et al., 2009; Todd et al., 2001). Bacterial DNA is cut with a frequently cutting restriction endonucleases (e.g., *Nci*I, *Eco*RI) resulting in hundreds of short fragments which are then separated by conventional agarose gel electrophoresis. The similarity of the generated DNA fragments patterns is used to differentiate the compared bacterial isolates and analyze the genetic relatedness of the strains (Busse et al., 1996). RFLP is often combined with a transfer of agarose‐separated fragments to the nitrocellulose or nylon membranes and their hybridization with one or more labeled specific probes (Swaminathan et al., 1996). Thus, only DNA fragments with the sequences specific to the sequences of the probes are detected which simplify the analysis (Li et al., 2009). The RFLP method has been commonly used to identify the small but specific variations in a sequence of DNA. The main advantage of RFLP analysis over PCR‐based protocols is that no prior sequence information, nor oligonucleotide synthesis, is required. However, the whole method is time‐consuming, requires a large amount of sample DNA, needs a suitable probe library, and therefore, currently it is not often used for typing of *L. monocytogenes* (Li et al., 2009).

A variant of the RFLP method, which uses probes directed to conserved domains of the 16S or 23S rRNA genes, is called ribotyping (Bingen et al., 1994). The main advantage of this approach over classical RFLP is that it enables analysis without prior knowledge of genomic DNA sequence because rRNA operons are universal (Li et al., 2009). Furthermore, a lower number of the DNA restricted fragments are produced; thus, the obtained ribotyping patterns are easier to evaluate (Bingen et al., 1994; Matloob & Griffiths, 2014).

#### 6.2.2 Pulsed‐field gel electrophoresis (PFGE)

Pulsed‐field gel electrophoresis is a molecular typing technique which is based on RFLP approach with the digestion of genomic DNA with a restriction endonuclease (or endonucleases) that recognizes specific sequences in the bacterial genome (Lopez‐Canovas et al., 2019; Wiedmann, 2002). The bacteria to be tested are cultured, harvested, and cells are washed in isotonic solution to maintain their integrity and then mixed with a low gelling temperature agarose (Lopez‐Canovas et al., 2019). The cells–agarose mix is applied to suitable mold plugs, then the bacterial cells are lysed to release DNA. Finally, restriction nuclease is added to digest of DNA into fragments, usually of 30–600 kb in size. The gel is subsequently subjected to electric field that periodically changes its direction to generate DNA banding patterns (Lopez‐Canovas et al., 2019).

The choice of restriction endonuclease is one of the most important factors in determining the PFGE banding pattern because the cleavage site of each enzyme is unique. For the differentiation of *L. monocytogenes* isolates, restriction enzymes such as *Apa*I, *Asc*I, and *Sma*I are most often used, either singularly or in combination of two different enzymes such as *Apa*I and *Sma*I, *Apa*I and *Asc*I, *Asc*I and *Sma*I, *Apa*I and *Not*I (Buchrieser et al., 1993; Jang et al., 2005; Park et al., 2016). Several other factors such as electric field strength, field angle and shape, agarose type and concentration, pulse time, ionic strength, and temperature have an influence on the obtained DNA pattern (Jang et al., 2005; Park et al., 2016).

Pulsed‐field gel electrophoresis has been used for many years in epidemiological investigations for subtyping many bacterial species, including *L. monocytogenes* (Buchrieser et al., 1993; Dalmasso & Jordan, 2014; Fugett et al., 2007; Jang et al., 2005; Lopez‐Canovas et al., 2019; Neves et al., 2008). The PFGE method seems to be a valuable choice for bacterial subtyping during outbreak investigations and it had been still considered as the gold standard in molecular epidemiology until the NGS era (Lopez‐Canovas et al., 2019; Wiedmann, 2002). The method has been standardized and the unique protocol for *L. monocytogenes* is publicly available (Swaminathan et al., 2001). Furthermore, a commonly used bioinformatics desktop software application BioNumerics developed by Applied Maths allows to produce high‐quality, reproducible results. Throughout PulseNet, a worldwide network of laboratories managed by the Centers for Disease Control and Prevention in Atlanta, Georgia, it is also possible to directly compare online the PFGE data generated in different laboratories.

### 6.3 Sequencing‐based typing methods

#### 6.3.1 Multiple locus variable‐number tandem repeat analysis (MLVA)

Multiple locus variable‐number tandem repeat analysis is another molecular method applied for subtyping of bacterial isolates (Lindstedt, 2005; Van Belkum, 2007). The analysis of the sequenced genomes revealed a high percentage of DNA that consisted of repeats, i.e., where some DNA sequences are present in multiple copies. These repetitive regions are either clustered in one specific area in the genome or dispersed throughout the whole genome (Van Belkum, 2007). Such DNA fragments are variable among bacterial strains with respect to the number or their individual primary structure and they are called “variable number of tandem repeat regions” (VNTRs). The MLVA method determines the number of tandem repeats, or copy units, at VNTR loci within the genome (Lindstedt, 2005). Briefly, the VNTR loci are first PCR amplified with flanking region‐specific primers and the PCR products are then subsequently separated according to their size by agarose gel electrophoresis or on an automated capillary DNA sequencer. The number of tandem repeats is assessed based on the size of the PCR products, and the string of alleles from multiple loci is used for the MLVA profile designation. This allows to assign to the strain a specific numerical code for a subspecies (Van Belkum, 2007). Each unique MLVA profile coded by a multidigit is assigned an MLVA type number, and it can be stored into a database for strain comparison and epidemiological studies (Nadon et al., 2017). There are several web‐based databases and analysis platforms that are designed for the MLVA results, e.g., MLVA‐Net hosted by the Pasteur Institute, France (www.pasteur.fr/mlva), MLVA bank curated by the University of Orsay, France (https://minisatellites.u‐psud.fr/MLVAnet/), or PulseNet International network (http://www.pulsenetinternational.org/protocols/Pages/mlva.aspx). The data available within these platforms allow to compare MLVA profiles of bacterial strains isolated worldwide and to determine distribution of MLVA types. The approach has been widely used for comparison and typing of *L. monocytogenes* isolates from different sources, including various foods (Chen et al., 2011; Løvdal et al., 2021; Lunestad et al., 2013; Martín et al., 2017; Miya et al., 2008; Murphy et al., 2007; Saleh‐Lakha et al., 2013). It has also been shown that MLVA had the capability to provide comparable or even slightly more discriminatory results when compared with other typing methods, including PFGE (Lindstedt et al., 2008; Murphy et al., 2007; Torresi et al., 2015; Volpe Sperry et al., 2008).

#### 6.3.2 Multi‐locus sequence typing (MLST)

The MLST method has been shown to be highly discriminatory for *L. monocytogenes* typing (Anwar et al., 2022; Salcedo et al., 2003). The approach is based on nucleotide sequences of fragments (loci) of housekeeping genes (usually seven) of approximately of 400–500 bp length of each which can be accurately sequenced on both strands using an automated DNA sequencer (Stessl et al., 2014). In MLST for *L. monocytogenes* typing, the following genes are usually amplified: *abcZ* (ABC transporter), *bglA* (beta‐glucosidase), *cat* (catalase), *dapE* (succinyl diaminopimelate desuccinylase), *dat* dat (D‐amino acid aminotransferase), *ldh* (lactate dehydrogenase), and *lhkA* (histidine kinase) (Kurpas et al., 2020; Ragon et al., 2008). Other gene combinations, including housekeeping and virulence genes which are under selection pressure and hence accumulate rapid sequence changes, have also been successfully used to obtain a higher level of discrimination than in classical MLST (Cai et al., 2002; Camargo et al., 2016; Jadhav et al., 2012; Salcedo et al., 2003; Wang et al., 2012). Such variants based on differences in nucleotide sequences of virulence and virulence‐associated genes is designed as multi‐virulence locus sequence typing (MVLST) (Jadhav et al., 2012). Following sequencing, the obtained DNA sequences present in the alleles at each of the seven housekeeping gene or other gene loci define the arbitrary allelic profile or sequence type (ST) (Enright & Spratt, 1999; Jolley & Maiden, 2014). Further, STs differing by no more than one allele from at least one other ST in the group are defined as clonal complex (CC) (Ragon et al., 2008). Since changes in the nucleotide sequences of housekeeping genes occur relatively slowly, the MLST method provides a good discriminatory power and is very suitable for strains typing and differentiation during epidemiological investigations (Cooper & Feil, 2004; Enright & Spratt, 1999). Another important advantage of MLST is that sequence data are unambiguous and the allelic profiles of isolates and the STs obtained in different laboratories can easily be compared to those stored in databases (e.g., https://pubmlst.org; www.mlst.net) and they can be queried via the internet (Jolley et al., 2004, 2018; Pérez‐Losada et al., 2013; Platt et al., 2006).

There are also online softwares (e.g., eBURST) for determination of the genetic relatedness between *L. monocytogenes* isolated from different sources or geographical regions as well as MLST‐maps to track the isolates of particular sequence types (Sabat et al., 2013). Furthermore, MLST is a relatively simple technique that can be readily reproduced and does not require access to specialized reagents or training, although it is cost‐related (Maiden, 2006; Sabat et al., 2013). However, it has been also observed that the MLST method was less discriminatory for *L. monocytogenes* isolates of serotype 4b which were better differentiated with PFGE (Chen & Knabel, 2007). On the other hand, the studies performed in China with 19 pathogenic *L. monocytogenes* isolated from food revealed that the MLST possessed the higher discriminatory potential than *Sma*I‐based PFGE since 19 and 17 subtypes were identified, respectively, although the obtained differences were not significant (discrimination index D.I. 0.990 vs. 0.976) (Jiang et al., 2008). Furthermore, the virulence genes (*actA*, *inlA*, and *inlB*) used for MLST had better discriminatory power than targeting the four (*betL*, *dat*, *sigB*, and *recA*) selected housekeeping genes only (D.I. 0.990 and 0.895, respectively) (Jiang et al., 2008). A comparable discriminatory potential of the MLST and PFGE methods has also been described by other authors (Henri et al., 2016; Jadhav et al., 2012; Zhang et al., 2004), although there are also information that MLST is a more powerful molecular tool for differentiation of *L. monocytogenes* isolates (Mohan et al., 2021; Revazishvili et al., 2004) or *vice versa* (Ariza‐Miguel et al., 2015; Hilliard et al., 2018; Wang et al., 2012). Nevertheless, the MLST approach was one of the most commonly used (and it is still applied) typing methods for *L. monocytogenes* in assessing the genetic variation in the bacterial population clusters and in studying molecular epidemiology of listeriosis before the NGS era (Anwar et al., 2022; Pérez‐Losada et al., 2013).

#### 6.3.3 Next‐generation sequencing (NGS)

Next‐generation sequencing, a DNA sequencing‐based approach which has revolutionized genomic studies, is a highly powerful molecular tool which allow to identify and characterize bacterial pathogens more rapidly and precisely than traditional methods, and can provide new insights into disease transmission, virulence, and antimicrobial resistance (Levy & Myers, 2016; Yohe & Thyagarajan, 2017; Zhong et al., 2021). The milestone in the NGS development was introduction of the pyrosequencing technology by the 454 Life Sciences company in 2005, where genomic DNA fragments hybridized to the surface of agarose beads, amplified, and then sequenced without any cloning step (Margulies et al., 2005). This high‐throughput method allowed the amplification and identification of billions of short‐sequencing reads in a single automatic run (Buermans & den Dunnen, 2014). Then, the Solexa company (currently Illumina) developed a pyrosequencer that uses glass‐attached oligonucleotides that are complementary to specific adapters previously ligated onto DNA library fragments. The sequences amplified with an isothermal polymerase are then subjected to sequencing by synthesis of the complementary strand and fluorescence‐based detection of reversibly blocked terminator nucleotides (Besser et al., 2018). Different Illumina instruments are offered, from MiniSeq and MiSeq with the low and mid sample throughput range of 0.3–15 Gb (giga base) to NextSeq, HiSeq, and NovaSeq instruments with much higher throughput, up to 6000 Gb, which, however, require additional automation for library preparation (Besser et al., 2018). Since then, a number of various NGS platforms using different sequencing technologies, which are able to perform sequencing of millions of small fragments of DNA at the same time, have been developed (Buermans & den Dunnen, 2014; van Dijk et al., 2014; Kanzi et al., 2020; Levy & Myers, 2016; Vincent et al., 2017). Sequencing approaches deliver by the main NGS players are either short‐read (100–400 bp) sequencing platforms (Illumina; Thermo Fisher) or long‐read (>500 bp) platforms (Pacific Biosciences and Oxford Nanopore Technologies). Regardless of the platform used, the general approach for a typical NGS run begins with genomic DNA extraction from test (e.g., bacterial) samples, library preparation involving DNA fragmentation either mechanically or enzymatically, ligation of adaptors, adaptor sequencing, template preparation, either by bridge amplification or emulsion PCR, and then final automated sequencing (Buermans & den Dunnen, 2014; Levy & Myers, 2016; Slatko et al., 2018). Each of the platforms has its own advantages and disadvantages related to accuracy, efficiency, and the cost. The whole process, from nucleic acid extraction through final sequence reporting, typically takes a minimum of 5–10 days (Petersen et al., 2020). Furthermore, highly advanced bioinformatics knowledge is required for data processing and analysis (Shendure & Ji, 2008).

After the development of the first‐ (the Sanger's chain termination method) and the second (pyrosequencing)‐generation sequencing approaches, which have been successfully used for several years and still are wildly used, a novel sequencing method based on nanopore technology, called the third‐generation sequencing, was introduced (Bleidorn, 2016; Gavrielatos et al., 2021; Kuleshov et al., 2014; Petersen et al., 2020; Rhoads & Au, 2015; Schadt et al., 2010). There are three main different platforms of this new method, both rely on very distinct principles but all are based on amplification of long‐read sequencing of native molecules (DNA or RNA) (Amarasinghe et al., 2020). The first approach, Single‐Molecule Sequencing in Real Time (SMRT) platform, was commercially released at the beginning of 2011 by Pacific Biosciences (Eid et al., 2009; Roberts et al., 2013). The method utilizes a sequencing‐by‐synthesis technology based on real‐time imaging of fluorescently labeled nucleotides which are synthesized along individual DNA templates. During the sequencing process, the fluorescence signals are activated by a laser as soon as a labeled dNTPs is incorporated into DNA, and then a camera system records the color and duration of the emitted light in real time (Xiao & Zhou, 2020). SMRT can very quickly generate very long reads of sequence (10–15 kb long) from single molecules of DNA. Although reads have a raw error rate of 10%–15%, several algorithmic techniques have been developed that can improve the per‐nucleotide accuracy to over 99.99% or more with sufficient coverage (Carneiro et al., 2012; Chin et al., 2013). The main limitation of the SMRT sequencing technology is a relatively high cost, especially when compared to second‐generation approaches.

The second variant of the third‐generation sequencing approach is the MinION platform which utilizes nanopores for sequencing and was commercially released in 2014 by Oxford Nanopore Technologies (Laver et al., 2015). The method is based on the passage of an ionic current across the flow cell during sequencing, and the different nucleotide bases are distinguished by the changes in current as they pass through the 2048 (MinION) or 12,000 (PromethION) individual nanopores incorporated into an electrically resistant membrane (Laver et al., 2015). The current in the nanopore is measured by a sensor several thousand times per second and is graphically presented as a plot. Finally, data processing is performed by the minKNOW software (van Dijk et al., 2014). The read lengths of the currently available MinION platform instrument are similar to those produced by Pacific Biosciences, and the accuracy of genomes sequenced using is over 99.95% (Loman et al., 2015). Furthermore, the obtained raw sequence data may be analyzed using cloud computing by the internet access (Bleidorn, 2016).

Another third‐generation sequencing platform was developed in 2012 by Illumina as Moleculo protocol and then marked as the Illumina TruSeq™ Synthetic Long‐Read technology (Kuleshov et al., 2014). Using this approach, up to10 kbp molecules of DNA are clonally amplified and barcoded before sequencing with a short read instrument with a high accuracy of ca. 99.99% (Kuleshov et al., 2014; Palomares et al., 2019). However, because TruSeq™ relies on long‐range amplification and the reads are synthetically generated, the available read lengths are shorter than obtained within other approaches (Palomares et al., 2019). Another disadvantage of this platform is that the receiving sufficient sequence coverage is rather expensive as compared to other platforms, e.g., SMRT sequencing (Almomani et al., 2020; McCoy et al., 2014).

Different platforms of NGS approaches are increasingly being used for *L. monocytogenes* molecular typing, epidemiological surveillance, outbreak investigations, and even in monitoring programs in food processing facilities in many countries (Hurley et al., 2019; Jackson et al., 2016; Jagadeesan, et al., 2019; Jagadeesan, Gerner‐Smidt, et al., 2019; Kwong et al., 2016; Moura et al., 2016, 2017; Schmid et al., 2014). Conventional molecular typing techniques, such as PFGE, MLVA, and MLST, which have been used for several years for the *L. monocytogenes* isolates differentiation, although still broadly utilized for clustering and epidemiological purposes, provide a lower resolution information compared to NGS. This modern typing method allows to determine the genomic diversity of *L. monocytogenes*, which is especially important for characteristics and differentiation of hypervirulent and persistent isolates, identification of potential sources of contamination, and assessment of putative pathogenic properties of isolates with different virulence genotype (Hurley et al., 2019; Jagadeesan, Baert, et al., 2019; Kurpas et al., 2020; Lachtara et al., 2021; Moura et al., 2016; Wieczorek et al., 2020). Therefore, the precise source of *L. monocytogenes* contamination tracking using NGS approaches is a crucial point during epidemiological investigations. This technique, together with newly developed bioinformatics tools, is currently routinely used during listeriosis outbreaks investigations in some countries (Jackson et al., 2016; Jagadeesan, Baert, et al., 2019; Kvistholm Jensen et al., 2016; Mäesaar et al., 2021; Moura et al., 2017; Ruppitsch, Prager, et al., 2015). One of these bioinformatic approaches widely utilized for subtyping of *L. monocytogenes* is core genome MLST (cgMLST) typing method based on assessment of allelic differences in 1748 genes, developed and validated at the Institute Pasteur, France (Moura et al., 2016). This subtyping scheme has been implemented in the BIGSdb software to provide free online access to perform the sequence analysis and allowing a standardized comparison with isolate databases for outbreak investigations and surveillance (Jolley & Maiden, 2010; Moura et al., 2016, 2017). However, this typing method is able to assess only differences present within the core genome which covers ca. 58% of the *L. monocytogenes* genome in relation to the number of the bacteria genes; thus, polymorphisms present in intergenic regions or in accessory genes are missing (Moura et al., 2016). The cgMLST was used to confirm the human isolates responsible for listeriosis in Germany lasting from 2012 to 2015 and showed that six strains with the identical PFGE patterns belonged to independent sequence cluster types (Ruppitsch, Pietzka, et al., 2015). Furthermore, thanks to the cgMLST analysis, the human cases could be traced back and the source of *L. monocytogenes* responsible for the outbreak was identified (Kleta et al., 2017). These and other results show that NGS technology, due to its high‐throughput capability and the development of new bioinformatics tools to analyze the obtained sequence data, is the most specific molecular tool for subtyping of microorganisms, including *L. monocytogenes* (Hurley et al., 2019; Jagadeesan, Baert, et al., 2019; Lachtara et al., 2021; Lüth et al., 2018, 2021; Moura et al., 2017; Orsi et al., 2021).

## 7 CONCLUSION

The reference methods for the detection of *L. monocytogenes* are based on bacteria culture and allow the recovery of this pathogen from different kinds of food and food production environments. These approaches are relatively simple, cheap, and do not require highly educated laboratory personnel. However, they are rather time‐consuming, although the introduction of chromogenic media significantly improved the *L. monocytogenes* identification process. Moreover, several alternative rapid and sensitive methods have been developed, but the obtained positive results must be confirmed by standard microbiological analyzes. Most of these quick techniques are based on DNA amplification (PCR, real‐time PCR), immunological principles (TRANSIA™ PLATE *Listeria monocytogenes*, VIDAS^®^ LMO2), or biosensor technology (Bio Electric Diagnostics). The rapid identification tests are usually of interest to food producers or food business operators because they are high sample throughput, cost‐effective, and may be used in food manufacture quality control programs. All these classical microbiological and rapid methods are continuously being developed and improved in order to provide higher sensitivity and specificity of *L. monocytogenes* detection.

Molecular biology‐based methods are currently the most rapid, sensitive, and effective in detection and differentiation of *L. monocytogenes* in foods; thus, they are widely used in laboratory identification of this bacterium. Such approaches are especially crucial in early detection of *L. monocytogenes* contaminated food which is important in prevention of the outbreaks of foodborne illness. Although molecular methods provide many advantages, there are still some limitations such as the need to use highly advance technology that are costly compared to conventional methods and require a well‐trained laboratory personnel. Combination of two or more *L. monocytogenes* detection methods, based on standard microbiological analyzes or alternative rapid approaches with molecular techniques, may improve the accuracy of detecting the pathogen in foods and complex food production environments. Such complex identification method should ideally be specific, sensitive, fast, simple, reproducible, cost‐effective, and able to distinguish between dead and live bacterial cells. Thus, further studies on the development of different combinations of various classical and molecular methods for *L. monocytogenes* identification in foods are required.

Classical microbiology‐based methods, rapid approaches, and molecular techniques are important for the routine testing of food and food production environments toward the presence or number of *L. monocytogenes*; however, they are not suitable for characteristics and typing of the bacterial isolates, which is crucial in the study of listeriosis outbreaks. For these purposes, several molecular‐based approaches have been developed which are characterized by high discriminatory power to distinguish the strains during epidemiological studies. Numerous methods for subtyping *L. monocytogenes* isolates are available and most of them are standardized, robust, reliable, and give reproducible results. Recently, whole‐genome sequence‐based techniques such as NGS have been developed and provided an opportunity to perform comparison between strains of the same species. These methods, due to their high‐throughput capability combined with the development of new bioinformatics tools to analyze the obtained sequence data, are already widely used in epidemiological studies during listeriosis outbreaks. The molecular investigations also provide a better understanding of already known and new virulence factors as well as pathogenic and antimicrobial resistance mechanisms of *L. monocytogenes* responsible for foodborne infections. However, there are still some limitations in these methods such as the need to use highly advanced and costly equipment and technology that is more expensive compared to conventional methods. Despite these limitations, the molecular typing methods, especially those based on next‐generation sequencing of the whole bacterial genome, are the future of routine testing and research on a better understanding of genetics, pathogenic potential, and epidemiology of *L. monocytogenes* as well as of human foodborne listeriosis cases and outbreaks.

## CONFLICT OF INTEREST

The authors declare no conflict of interest.
